# Bioinformatics tools in predictive ecology: applications to fisheries

**DOI:** 10.1098/rstb.2011.0184

**Published:** 2012-01-19

**Authors:** Allan Tucker, Daniel Duplisea

**Affiliations:** 1 School of Information Systems, Computing and Maths, Brunel University, Uxbridge, Middlesex UB8 3PH, UK; 2 Fisheries and Oceans Canada, Institut Maurice-Lamontagne Mont Joli, Quebec, Canada

**Keywords:** bioinformatics, Bayesian networks, classification, dynamic models, fisheries management

## Abstract

There has been a huge effort in the advancement of analytical techniques for molecular biological data over the past decade. This has led to many novel algorithms that are specialized to deal with data associated with biological phenomena, such as gene expression and protein interactions. In contrast, ecological data analysis has remained focused to some degree on off-the-shelf statistical techniques though this is starting to change with the adoption of state-of-the-art methods, where few assumptions can be made about the data and a more explorative approach is required, for example, through the use of Bayesian networks. In this paper, some novel bioinformatics tools for microarray data are discussed along with their ‘crossover potential’ with an application to fisheries data. In particular, a focus is made on the development of models that identify functionally equivalent species in different fish communities with the aim of predicting functional collapse.

## 1. Introduction

Bioinformatics has revolutionized the way we analyse molecular biological data. Owing to the explosion in data collection and storage made available since the dawn of parallel sequencing, there has been a demand for specialist techniques to analyse and model data such as microarray experiments, which measure the expression of thousands of genes simultaneously. The advance of research in fields including machine learning [1], data mining [2] and intelligent data analysis [3,4] has resulted in many novel tools for the analysis of such data. In bioinformatics, techniques such as clustering were initially extremely popular for identifying groups of genes with similar expression profiles [5,6]. This allowed biologists to identify the function of previously unknown genes through ‘guilt by association’. It also allowed these groups to be treated as single modules [7,8] in order to reduce the massive number of variables when building models for prediction. Classification of disease outcome [9] has also been very popular with many approaches being developed, including methods to identify relevant biomarkers through feature selection [10]. Modelling time-series microarray data has been useful in understanding the underlying dynamics of microarray time-series, and cell-cycle data have been a popular topic of study [11]. One particular development in these areas is the adoption of graph-based models in the form of genetic regulatory networks (GRNs) [12,13]. These approaches allow biologists to explore the complexities of gene interaction on a large scale and therefore take a *systems* approach to modelling.

In contrast, ecological data analysis has been rather less explorative to date when compared with bioinformatics and systems biology. There are of course exceptions, and in the study of Hochachka *et al.* [14] a discussion of the potential of using data-mining techniques is explored for situations where there is little or no prior knowledge about a system. In this paper, we investigate the cross-over potential of techniques used in bioinformatics, such as feature selection, classification, Bayesian networks (BNs) and in particular an adaptation of an algorithm that we previously developed for exploiting the availability of multiple datasets. This is applied to fisheries data in order to identify species that perform similar functional roles in different fish communities. These equivalent species are used to predict functional collapse in their respective regions through the use of dynamic Bayesian models with latent variables.

In the remainder of this section, BNs are introduced in the context of bioinformatics research, and recent relevant work on specialist bioinformatics techniques that have cross-over potential is discussed. The use of these techniques applied to ecological data is also discussed with a focus on fisheries. In §2, the fisheries data and the ‘functional equivalence’ algorithm are described. Results in §3 demonstrate how models learned from data in one region can be used to identify and predict the biomass of ‘functionally similar’ species and as a result, the functional collapse in other regions. Finally, the use of the techniques explored in this paper (namely, BNs for feature selection and classification, the functional equivalence algorithm and dynamic models with latent variables) are discussed in §4 in terms of the wider ecological literature.

### (a) Bayesian networks for bioinformatics

**Figure 1.**
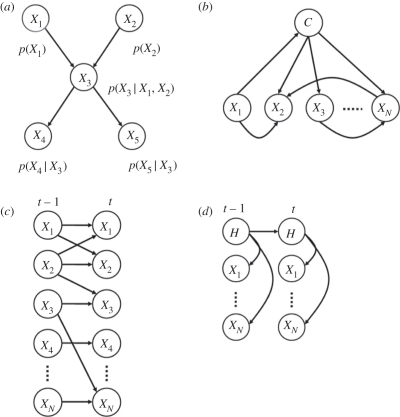
(*a*) A Bayesian network (BN) that encodes a joint distribution using a graphical structure and local conditional distributions. Links between variables represent conditional independences. (*b*) A BN classifier where *C* denotes a class node to predict. (*c*) A dynamic BN where nodes represent variables at a point in time and (*d*) a hidden Markov model, where *H* denotes an unmeasured (hidden or latent) variable.

BNs have become a popular method for computational modelling of GRNs from microarray expression data [15–17]. A BN describes the *joint distribution* (which is a way of assigning probabilities to every possible outcome over a set of variables, *X*_1_ … *X*_*N*_) by exploiting conditional independence relationships represented by a directed acyclic graph (DAG). See figure 1*a* for an example of BN with five nodes. Each node in the DAG is characterized by a state which can change depending on the state of other nodes and information about those states propagated through the DAG. This kind of inference facilitates the ability to ask ‘what if?’ questions of the data by entering evidence (changing a state or confronting the DAG with new data) into the network, applying inference and inspecting the *posterior distribution* (which represents the distributions of the variables given in the observed evidence). For example, one could ask, what is the probability of seeing gene A ‘switch on’ (through high expression) given that we have observed a low expression in genes B and C?

There are numerous ways to infer both network structure and parameters from data. Constraint-based approaches such as the PC [18] and IC* [19] algorithms both work by applying independence tests between variables and building networks that reflect these independences. However, these do not scale well for high-dimensional datasets and are prone to getting stuck in local minima. Search-and-score methods to infer BNs from data have been used frequently in learning GRNs [15]. These methods involve performing a search through the space of possible networks and scoring each structure. A variety of search strategies can be used [20–23]. BNs are capable of performing many data analysis tasks including feature selection and classification (performed by treating one node as a class node and allowing the structure learning to select relevant features [24] (figure 1*b*)). Modelling time series can be achieved by using an extension of the BN known as the dynamic Bayesian network (DBN) [25,26], where nodes represent variables at particular time slices (figure 1*c*). Closely related to the DBN is the Hidden Markov Model (HMM) which models the dynamics of a dataset through the use of a latent variable [27]. This latent variable is used to infer some underlying state of the series and through an autoregressive link that can capture relationships of a higher order (figure 1*d*).

BNs offer a natural mechanism for incorporating prior knowledge relating to the network structure through informative structure priors [28]. There has been substantial work in using priors to build more robust GRNs. Steele *et al.* [22] use concept profiles learned from abstracts in the biological literature (Medline) to bias BN learning algorithms and found that lesser studied systems generally gain more from updating priors with new data. Imoto *et al.* [29] use energy functions to incorporate prior knowledge sources including literature-based knowledge extracted from regulatory interactions that are recorded in the Yeast Proteome Database (YPD). In the study of Werhli & Husmeier [30], the approach was extended to multiple sources of prior knowledge, applied to combining protein–protein interactions and pathways from KEGG (Kyoto Encyclopedia of Genes and Genomes) with expression data.

### (b) Consensus and functional models

Comparing apparently similar multivariate datasets is often problematic owing to differences in collection methods. Such often is the case for microarray data which have methodological and laboratory dependencies [31] and similar issues occur with ecological community data collected for different systems. Though data normalization is the logical solution to such problems, it is neither straightforward nor a complete solution [32,33]. A post-learning aggregation framework called *consensus BNs* was developed for microarray datasets [34] to overcome some of these issues by combining datasets generated by different platforms, research groups and laboratories without requiring normalization. In this framework, learnt models that are generated from each dataset are aggregated, producing a combined model that represents prominent features which occur in all, or a subset of, the individual dataset models. The problem with this approach is the need to pre-select higher quality datasets to prevent the ‘dumbing down’ of networks from lower quality data resulting in an ‘average’ network rather than a ‘best-of’. A reliable method to identify these higher quality datasets prior to the consensus algorithm was found to be the *predictive accuracy* of models learned from one dataset and tested on other available independent sets [35]. This approach resulted in consensus models that were consistently more parsimonious to biologically validated networks and was extended by Anvar *et al.* [36,37]. It is this idea of exploiting independent datasets that shapes the work in this paper.

In summary, the success of bioinformatics methods such as feature selection, classifiers and HMMs has led to many novel discoveries including the identification of biomarkers, the prediction of disease outcome and GRNs built at a systems level. What is more, the exploitation and integration of multiple data sources allow more robust regulatory mechanisms to be identified and predictions to be made across very different platforms and organisms. We now demonstrate the transfer of some of these methods to ecological data with an application in fisheries.

### (c) Fisheries and ecoinformatics

**Figure 2.**
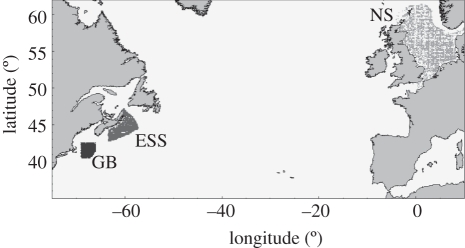
Georges Bank (GB), the East Scotian Shelf (ESS) and the North Sea (NS). The focus of the empirical analysis.

In this paper, the focus is on the application of bioinformatics techniques described in §1 to biomass data from Georges Bank (GB in figure 2), the East Scotian Shelf (ESS) and the North Sea (NS) between the years 1960 and 2007. Data are typically noisy and there are similar data quality issues as found with many microarray datasets. There are also multiple studies carried out throughout the world and prior expertise available much similar to bioinformatics datasets. For example, food webs that describe predator–prey and competitor species are available. Some of these are more detailed than others and may include the results of stomach surveys [38], where the diet of specific species can be determined.

The experiments carried out in this paper focus on cod biomass. Some spectacular collapses in fish stocks have occurred in the past 20 years but the most notable is the once largest cod stock in the world, the Northern cod stock off eastern Newfoundland, which experienced a 99 per cent decline in biomass. Cod, unfortunately, is not alone and there are stocks of various species that have been reduced to only a small percentage of stock sizes in recent history. Much of this effect is due to direct mortality on fish through fishing and subsequent indirect effects and weak linkages with other species. Some of these regions may have moved to an ‘alternative stable state’ or experienced a ‘regime shift’ and are unlikely to return to a cod-dominated community without some chance event beyond human control [39].

Different species may have similar functional roles within a system depending on the region. For example, one species may act as a predator of another which regulates a population in one location, but another species may perform an almost identical role in another location. If we can model the function of the interaction rather than the species itself, data from different regions can be used to confirm key functional relationships, to generalize over systems and to predict impacts of forces such as fishing and climate change. The approach concerns functional network topology and avoids the necessity of describing the specifics of network nodes. For example, the ‘wasp waist’ (WW) is a common structure present in many temperate and boreal fish community food webs [40]. The WW functional structure is characterized by few or just one mid-trophic level species preying upon several lower trophic level species, while several high trophic species prey upon the mid-trophic level species. In this way, energy flow from low to high trophic level species is constricted at the mid-trophic level species analogous to a WW. These WW species exert undue influence on aquatic community structure by top-down control of lower trophic levels through predation and bottom-up control of higher trophic levels by restricting energy flow. The WW effect is found in populations in the northwest Atlantic and the northeast Atlantic. This functional structure is identical in the two regions but the species involved are different (in the northwest Atlantic one of the WW is the capelin and in the northeast one is known to be the sand eel).

This focus on critical sub-systems through the exploration of functionally equivalent species across different populations is a novel approach to fish population modelling. This approach to modelling fish populations will explore functional relationships (such as predator, prey and WW) that are generalizable between different oceanic regions allowing more robust models to be built and predictions to be made about future biomass. There is some research into using BNs for ecological modelling [41] and in particular for modelling fish populations [42,43]. There is also considerable literature on integrating heterogenous data within the data-warehousing community including the environmental data [44], but no exploration of integrating or comparing different variables under a single function as we do with species. In the study of Thrush *et al.* [45], functions are explored by investigating weights in hidden nodes of neural network models. Here, we focus on DBNs with latent variables that can be used in conjunction with human expertise to predict functional collapse in different dynamic systems.

A number of questions are posed based upon fish interaction:

— Can we use bioinformatics-style analysis (in particular, feature selection) to identify species that are relevant to some event such as cod functional collapse?
— Can we model the temporal and dynamic nature of fish interactions?
— Can we identify species in different oceans that perform similar functions, and therefore predict functional collapse in their respective regions?

Techniques such as those described in §1 will be employed to answer these questions within a BN framework. In particular, a novel algorithm—the *functional equivalence*—search is introduced to make inferences between the different geographical systems.

## 2. Material and methods

### (a) Data description

GB fish community data come from the National Marine Fisheries Service autumn multi-species trawl survey from 1963 to 2008. About 80 randomly selected stations were sampled on GB each year and annual averages of biomass of each species were calculated and used in this analysis. About 220 have been caught in the survey but most infrequently and with low statistical power; therefore, analyses were confined to a subset of 39 species filtered from the dataset for which we have confidence in their quantitative estimates of abundance each year. ESS and NS data were collected via a similar methodology as on GB and this resulted in subsets of 34 and 45 species, respectively. The sources of these datasets are outlined in the acknowledgements of this paper.

GB is a relatively small productive fishing bank historically supporting large catches of common groundfish such as cod and haddock and also with a very valuable sea scallop fishery. Fish on GB tend to have ideal growing conditions and mature quickly. GB is relatively self-contained with deep channels to the northeast and ocean currents containing waters on the bank giving the region a distinct character. However, the GB community does have seasonal migrants such as mackerel and dogfish which affect the community. Drastic changes occurred on GB in the late 1980s, where groundfish were much less abundant. We have termed 1988 as the collapse year for GB. The ESS, though geographically not far from GB is a much different system with lower productivity, diversity and more open to both the northwest and the southwest biologically and oceanographically. A key characteristic of the ESS is the presence of a small sandy arc 200 km offshore called Sable Island, which is the largest grey seal breeding colony in the world and has been growing exponentially since the mid-1980s. The ESS showed drastic declines in cod and some other groundfish in the early 1990s to almost undetectable levels. We consider 1992 to be the collapse year for ESS. The NS is a shallow warm sea with high fish community diversity and productive multi-species fisheries. The NS has supported very large groundfish and pelagic fisheries and despite extremely high fishing pressure, it is difficult to see a sudden change in the system that might be termed a collapse as seen in GB and ESS. The NS fish community always seems to respond positively to curtailment of fishing effort, while the equivalent is not true for GB and ESS.

### (b) Experiments

The experiments undertaken in this paper involve applying classification. This involves the prediction of a pre-selected variable (here functional collapse) based on the values of other variables (here species biomass). Feature selection is used to identify the relevant species for optimal classification. There are two approaches to feature selection: filter selection that simply scores variables (species) independently, and wrapper selection that builds models and selects combinations of variables (thus identifying interactions between them). These experiments adopt the BN classifier approach, where the class node is a binary variable that represents functional collapse in GB. The K2 search algorithm [20] is used to build the BN classifiers. This involves a greedy search technique where links are incrementally added to an initially unconnected graph and scored using the metric given in equation (2.1), where *n* is the number of nodes, *F*_*ijk*_ is the frequency of occurrences in the dataset that the node *x*_*i*_ takes on the value *vik* (where there are *r*_*i*_ possible instantiations) and the parent nodes *π*_*i*_ take on the instantiation *w*_*ij*_ (where there are *q*_*i*_ possible instantiations). This metric is based on equation (2.2), which calculates the probability of observing a structure *G* and a set of data *D*, *p*(*G*,*D*), where *c* is a constant prior probability *p*(*G*). For simplicity, we assume a step change in functional structure in 1988 for GB data and 1992 for ESS. Further work will explore using hidden variables with more states and continuous variables to explore intermediate stages prior to collapse. A bootstrap [46] approach is employed to repeat the following 1000 times:

1. Score each species using the likelihood score given in equation (2.1) and take the mean over the bootstrap. This is known as *filter* feature selection [10] and scores each variable independently.
2. Learn BN structure with the (greedy) K2 algorithm and score the proportion of times that links are associated with the class node during the bootstrap (the confidence). This is known as *wrapper* feature selection [10] and scores each variable by taking into account their interaction with other variables through the use of a classifier model.

2.1
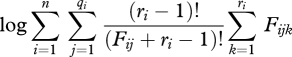
2.2
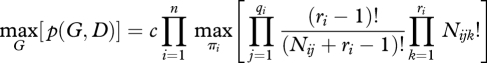
and2.3
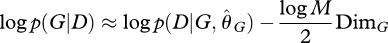


We rank species based upon these two feature selection approaches and examine their relevance to functional collapse in GB. In order to explore the functionally equivalent species in the NS and ESS data, we use species identified using feature selection from GB in conjunction with the *functional equivalence* search algorithm (which is fully documented in algorithm 1). This is applied to both the NS and ESS to identify equivalent species. Finally, we use dynamic models, specifically DBNs (as seen in figure 1*d*) but with a single dynamic hidden variable to identify functional collapse. These networks are built from the GB data (using the REVEAL algorithm [47], which is a greedy search applied to DBNs) to predict cod biomass and functional collapse (using the hidden variable).

The functional equivalence algorithm uses a simulated annealing approach [48] to search for an optimal combination of variables that fit the given function. This is where a random allocation of selected variables is initialized and scored. Within each iteration, a single replacement is made to the selected variables and the new selection is scored. Here, we demonstrate the approach using a BN model, where the given function is in the form of a predefined BN structure, *BN*_1_, and set of variables, *vars*_1_ that is parametrized from a dataset, *data*_1_. This model is then used to search for the variables in another dataset, *data*_2_ that fits best. The algorithm gives as output the set of variables that best fits the given model. We use the Bayesian Information Criterion which penalizes overly connected networks to avoid overfitting. It is given in equation (2.3), where *M* is the number of samples, *Dim*_*G*_ is the dimension of the model, and 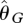 is the maximum-likelihood estimate of the parameters. The first term is essentially the log-likelihood and the second is a penalty for model complexity. We set iterations = 1000 and *t*_start_ = 1000 as these were found through experimentation to allow convergence to a good solution.

**Table:** **Algorithm 1.** The functional equivalence search algorithm.

Input: *t*_*start*_, *iterations*, *data*_1_, *data*_2_, *vars*_1_, *BN*_1_
Parametrize Bayesian Network, *BN*_1_ from *data*_1_
Generate randomly selected variables in *data*_2_: *vars*_2_
Use *vars*_2_ to score the fit with selected model *BN*_1_ using equation 2.2: *score*
Set *bestscore* = *score*
Set initial temperature: *t* = *t*_*start*_
**for***i* = 1 to *iterations***do**
Randomly replace one selected variable in *data*_2_ and rescore using equation 2.2: *rescore*
*dscore* = *rescore* − *bestscore*
**if***dscore* ≥ 0 OR *UnifR and* (0,1) < exp^(*dscore*/*t*)^**then**
*bestscore* = *rescore*
**else**
Undo variable switch in *vars*_2_
**end if**
Update the temperature: *t* = *t* × 0.9
**end for**
Output: *vars*_2_

## 3. Results

**Figure 3.**
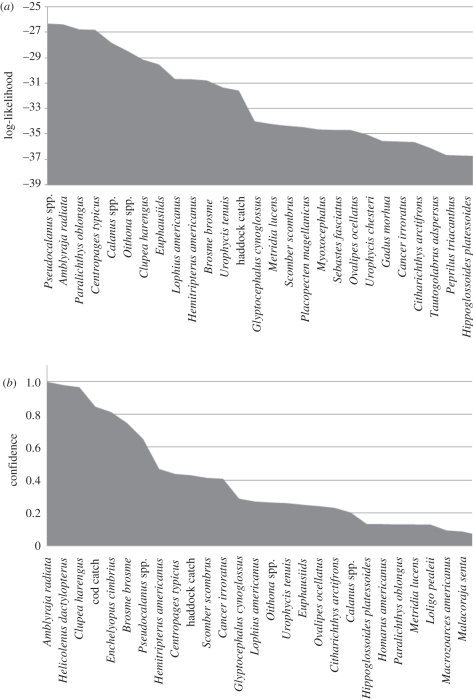
Features selected from GB data using a bootstrap on (*a*) filter selection using log-likelihood and (*b*) a Bayesian network classifier wrapper.

Figure 3 displays the rankings for filter and wrapper feature selection for differentiating between pre- and post-functional collapse in GB (1988). From both feature selection approaches, it is clear that there are a relatively small number of key players in this collapse and these are known to be involved with cod. For example, the likelihood approach strongly implicates two zooplankton species (*Calanus* and *Pseudocalanus*) as key to the functional collapse and it is known from other sources that there were relatively large changes then [49], and these changes can have bottom-up effects which affect species such as cod [50]. Herring (*Clupea harengus*) was also identified as a key species and its abundance changes in the late 1980 may have changed the predation environment of juvenile cod whose recruitment to adult stages may, in some systems, be significantly controlled by herring abundance [51]. Thorny skate (*Amblyraja radiata*) became more abundant at the time of the cod collapse on GB and although some attribute this to an ecosystem regime shift [52] others attribute this to immigration from the ESS [53].

**Figure 4.**
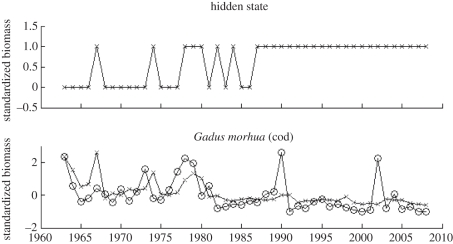
The fit for the model trained on GB data along with the associated discovered hidden state. The series marked with crosses denote the predicted biomass and hidden state as opposed to the observed biomass denoted by circles.

Using the higher ranking species from figure 3, a DBN model was built with a hidden node using the REVEAL algorithm (see §2) to confirm how predictable both cod biomass and the unobserved functional collapse were from the related species. Figure 4 plots these results and shows that a reasonable fit to the GB data is achievable. What is more, the hidden state identifies a noisy underlying process which appears to stabilize somewhere in the late-1980s correlating with the expected functional collapse.

**Figure 5.**
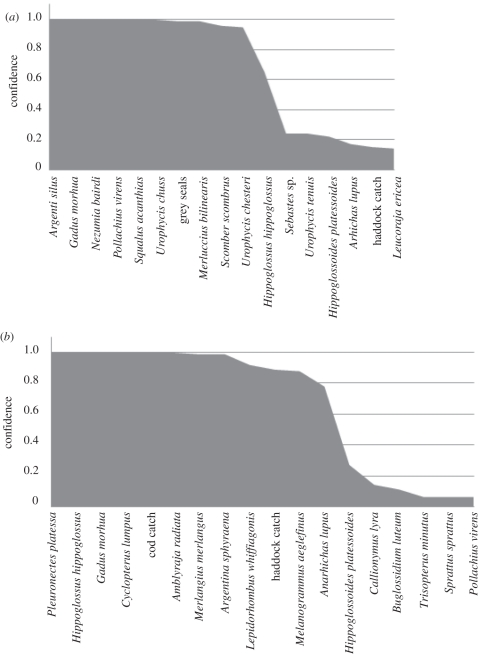
Functionally equivalent species to those selected from GB data identified using the functional equivalence algorithm. (*a*) Shows the equivalent species in the ESS and (*b*) shows the species in the NS.

The confidences resulting from the Bayesian wrapper method applied to GB showed a quick decline with species rank, such that thorny skate was the most important species implicated in the decline. When this structure is imposed on the ESS and NS using the functional equivalence search, a small number of functionally equivalent species are identified in both the ESS and the NS with high confidence (figure 5). An interesting thing to note was the species/processes on GB, where the change in the community seemed to be captured by changes in two zooplankton species while in the ESS and the NS, there was no strong indication of zooplankton changes that accompanied fish community change.

Perhaps, the most striking feature of the functional equivalence applied to the ESS is the presence of many deepwater species such as argentine (*Argentina sphyraena*), grenadier (*Nezumia bairdi*) and hakes (*Merluccius bilinearis*). Surprisingly, cod was not implicated in the ESS collapse despite the fact that cod were a highly targeted species prior to collapse. The inclusion of grey seals is also expected as they were implicated in the decline and lack of recovery of many groundfish stocks on the ESS. The largest breeding colony of grey seals in the world is located on Sable Island in the middle of the ESS.

The presence of coldwater-seeking deepwater species on the ESS could be an indication of the water cooling that occurred on the ESS in the late-1980s and early-1990s, which also led to increases in coldwater shrimp and snow crabs. Furthermore, though grey seals increased in abundance at the same time, grey seals are not deep divers and if the deepwater species remained in the shelf basins and slope water, they would be less susceptible to grey seal predation than would cod.

In the NS, most of the selected species are commercially desirable and some experienced large declines in biomass in this period, though the nature of the species is not dissimilar to GB when compared with ESS, which showed the appearance of some qualitatively very different species. Catch of haddock and cod appeared to be important in the NS while commercial fish catch seemed less important on the ESS. These factors combined might suggest that catch is one of the most important factors driving change in the NS, while on the ESS, it may be that other factors lead to fundamental changes in the fish community composition.

**Figure 6.**
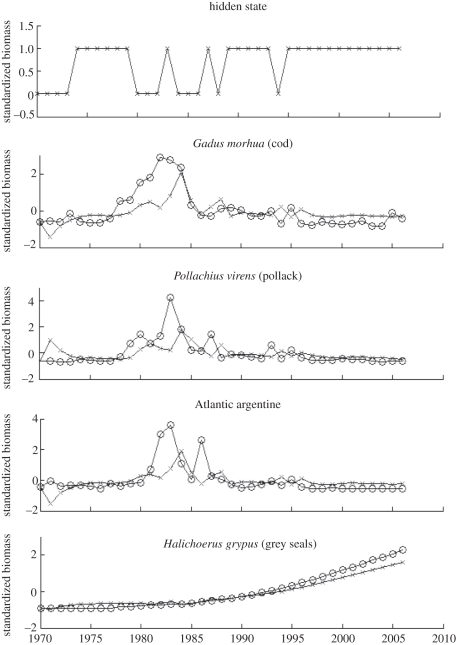
One-step ahead prediction of cod using DBN model trained on GB data and mapping to equivalent species in the ESS (identified using the functional equivalence algorithm along with the associated discovered hidden state). The series marked with crosses denote the predicted biomass and hidden state as opposed to the observed biomass denoted by circles.

The final set of results explore how well the functionally equivalent species can predict future biomass and the underlying state of the geographical system. Figure 6 documents these results for the selected functionally equivalent species for ESS (using the DBN trained on GB data and then mapped on equivalent species on ESS). The prediction of many of these species was surprisingly good, with close fits to the observed data. This is impressive considering that the model was parametrized using biomass data from different species in GB. For example, the model predicts the increase in seal numbers year after year based upon parameters determined on the relationship between cod catch and other species in GB. What is more, the hidden state inferred from the predicted data resembles very much what was observed in terms of functional collapse. While the state fluctuates in the period up to the late-1980s/early-1990s, in the period after the collapse the state becomes very stable. This further adds credence to the conclusion that the selected species are indeed key to the functional collapse of cod in the ESS.

**Figure 7.**
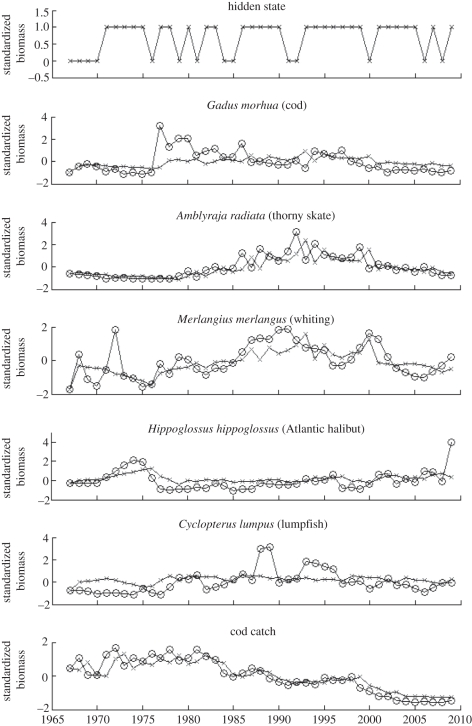
One-step ahead prediction of cod using DBN model trained on GB data and mapping to equivalent species in the NS (identified using the functional equivalence algorithm along with the associated discovered hidden state). The series marked with crosses denote the predicted biomass and hidden state as opposed to the observed biomass denoted by circles.

The same analysis was applied to identifying functionally equivalent species in the NS and testing them for prediction of biomass and identifying changes in the underlying state. Figure 7 illustrates the results. Firstly, note that the hidden state does not appear much less stable than in the ESS results. Rather than identifying no change in state (as was expected as no collapse has been observed), the hidden variable appears to have fitted the states to some noise process that fluctuates throughout the series. This could be due to the hidden state capturing the functional collapse successfully, which is the most influential predictive feature of cod in the ESS dataset, whereas the prediction of cod in the NS is more complex due to the lack of any collapse.

## 4. Discussion

Since the large-scale fisheries collapses in many different regions globally in the late-1980s and into the 1990s, there has been a search for causal mechanisms (e.g. [54]). This research has included studies of fisheries on species [55] as well as indirect effects of modifications of food webs and functional structure [56]. They have included simulation studies on functional structures in food webs [54,57], development of static functional structures through covariance techniques [58] or summaries of complicated multivariate data to examine overall temporal trends [59]. The present use of machine-learning techniques and BNs is another method applied to the the problem.

The use of bioinformatics techniques in this paper is unique because it exploits functional equivalence between different datasets and uses the identified species in conjunction with a dynamic model that uses latent variables to predict functional collapse (and future biomass). The recognition of a latent variable is important in fish community change studies of this nature because it allows causes of change which are not purely found within the constrained model structure. This is very different from mass balance model approaches whose fitting is conditioned completely upon the model structure. The latent variable therefore may partially represent something external to the fish community such as oceanographic conditions. We intend to explore this further by using data of likely factors such as temperature, nutrients and fishing mortality. Changes in conditions external to the fish community may be responsible for collapse in GB and ESS. The longer runs of similar estimates for the hidden state compared with NS could suggest different processes occurring there. Oceanographic conditions are a contender for ESS. For GB, what is occurring is less clear. NS, being highly exploited but shallow and dynamic, may naturally be more variable and able to cope with disturbances that would send the other two systems into collapse. Further work is warranted and exploration of other processes such as system variability before and after collapse [60] may prove to be useful predictors of collapse.

BN models also facilitate the direct incorporation of expertise into the structures and parameters. While this has not been explored fully here (the use of food webs have been used mostly for validation), using informative priors in the network models based upon available expertise will be investigated. The modelling approach also differs from other methods in how correlative structures, which are assumed to represent causal functional relationships, discovered in one system can be imposed upon another system. The components of the other system which best fit these structures can then be found in other systems. The topology of the BN allows us to explore these structures explicitly and a follow-up study will explore them prior to and after suspected regime changes. Though most ecosystem studies recognize the functional relation approach between species, most cannot deal with it in as pure a sense. Essentially, what this approach assumes is that there are only a few ways for similar ecosystems to organize themselves functionally even though the components may have different qualities; our analysis suggests that there may be similar ways to collapse. This can provide real insights into why fished ecosystems collapse and why they sometimes do not recover when a perturbation stops. Most importantly, it may give us an insight into signs of an imminent collapse perhaps while there is still time to prevent it.
